# Supplementary material for: ZNF217 promotes receptor tyrosine kinase plasticity and AXL-ERK dependency in ovarian cancer
Source: bioRxiv. 2026 Sep 14:2026.09.11.750913. Preprint. [Version 1] doi: 10.64898/2026.09.11.750913 (PMC13596211; doi:10.64898/2026.09.11.750913)
Supplement: Supplementary file 1 [file media-1.pdf]

SUPPLEMENTAL TABLES AND TABLE LEGENDS

Target	Source	Catalog number	Dilution
ERBB2	Proteintech	18299-I-AP	1:1000 (WB)<br>1:300 (IF)
ZNF217	Invitrogen	720352	1:1000
ERBB3	Cell signaling	12708S	1:1000 (WB)
EGFR	Proteintech	18986-I-AP	1:1000 (WB)
pERB2(Tyr877)	Proteintech	82762-11-RR	1:1000 (WB)
p-AKT(S473)	Proteintech	66444-1-IG	1:1000 (WB)
p-AKT(T308)	Proteintech	81232-10-RR	1:1000 (WB)
PDK1	Proteintech	18262-1-AP	1:1000 (WB)
AKT	Proteintech	10176-2-AP	1:1000 (WB)
PTEN	Proteintech	22034-I-AP	1:1000 (WB)
FGFR1	Proteintech	60325-1-IG	1:1000 (WB)
FGFR4	Proteintech	81069-1-RR	1:1000 (WB)
AXL	Proteintech	84659-1-RR	1:1000 (WB)
p-AXL(Tyr779)	Proteintech	82816-4-RR	1:1000 (WB)
ERK 1/2	Proteintech	11257-I-AP	1:1000 (WB)
pERK1/2	Proteintech	80031-1-RR	1:1000 (WB)
Beta-Actin	Sigma	A228	1:10000 (WB)
FITC-anti rabbit	Proteintech	SA00003-2	1:600 (IF)

Table S1. List of antibodies, their source and dilutions used in this study.

SUPPLEMENTAL FIGURES AND FIGURE LEGENDS

Fig. S1

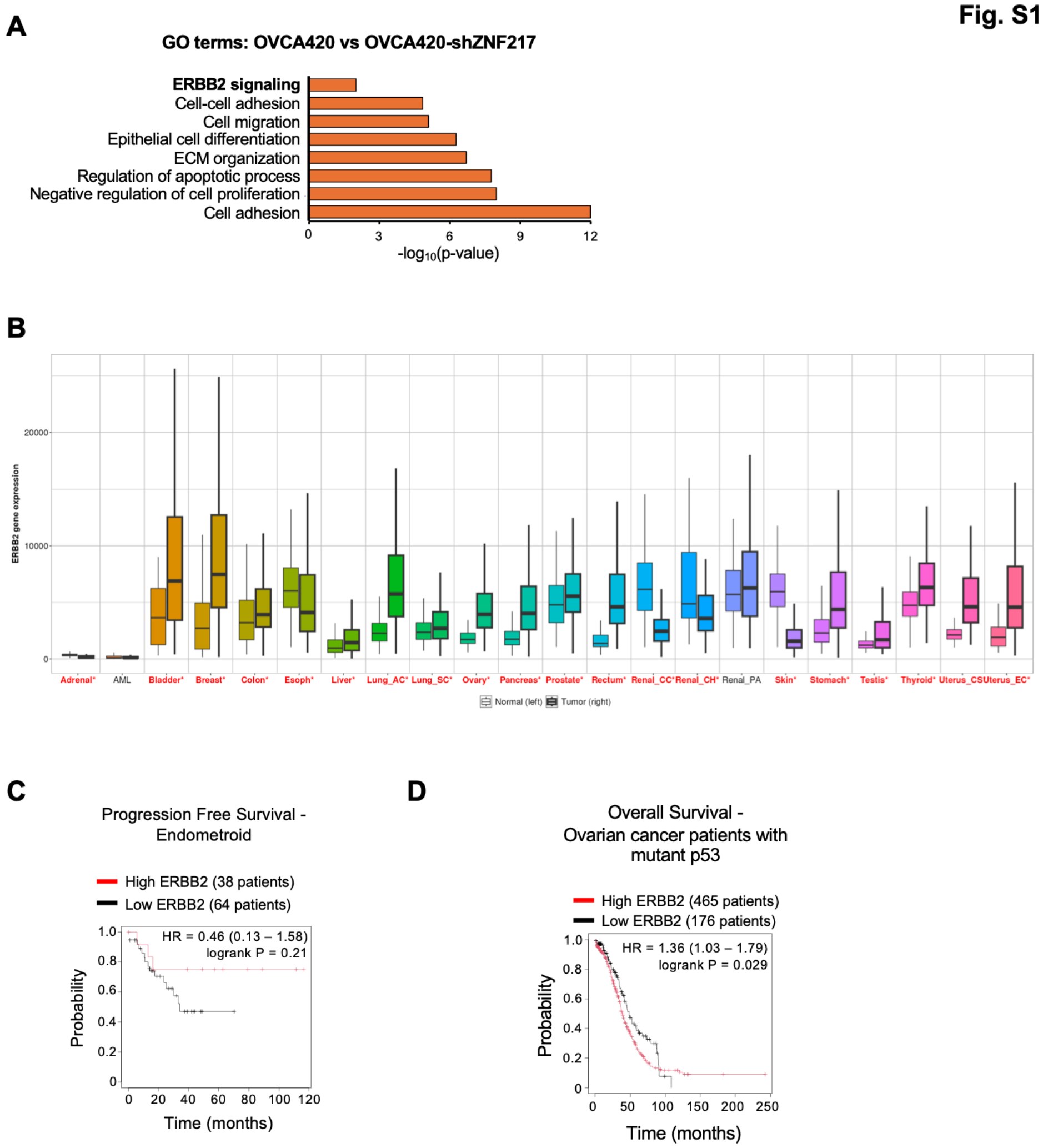

Figure S1. ERBB2 is overexpressed in ovarian cancer and associated with poor prognosis. **(A)** Gene ontology terms that are altered upon ZNF217 knockdown in OVCA420 cells. **(B)** ERBB2 expression is altered in many cancers including ovarian cancer. Data retrieved from TNMplot. **(C)** ERBB2 overexpression does not impact the progression free survival (PFS) in endometroid ovarian tumors. **(D)** ERBB2 overexpression causes a reduction in overall survival (OS) in mutant p53 driven ovarian cancer.

Fig. S2

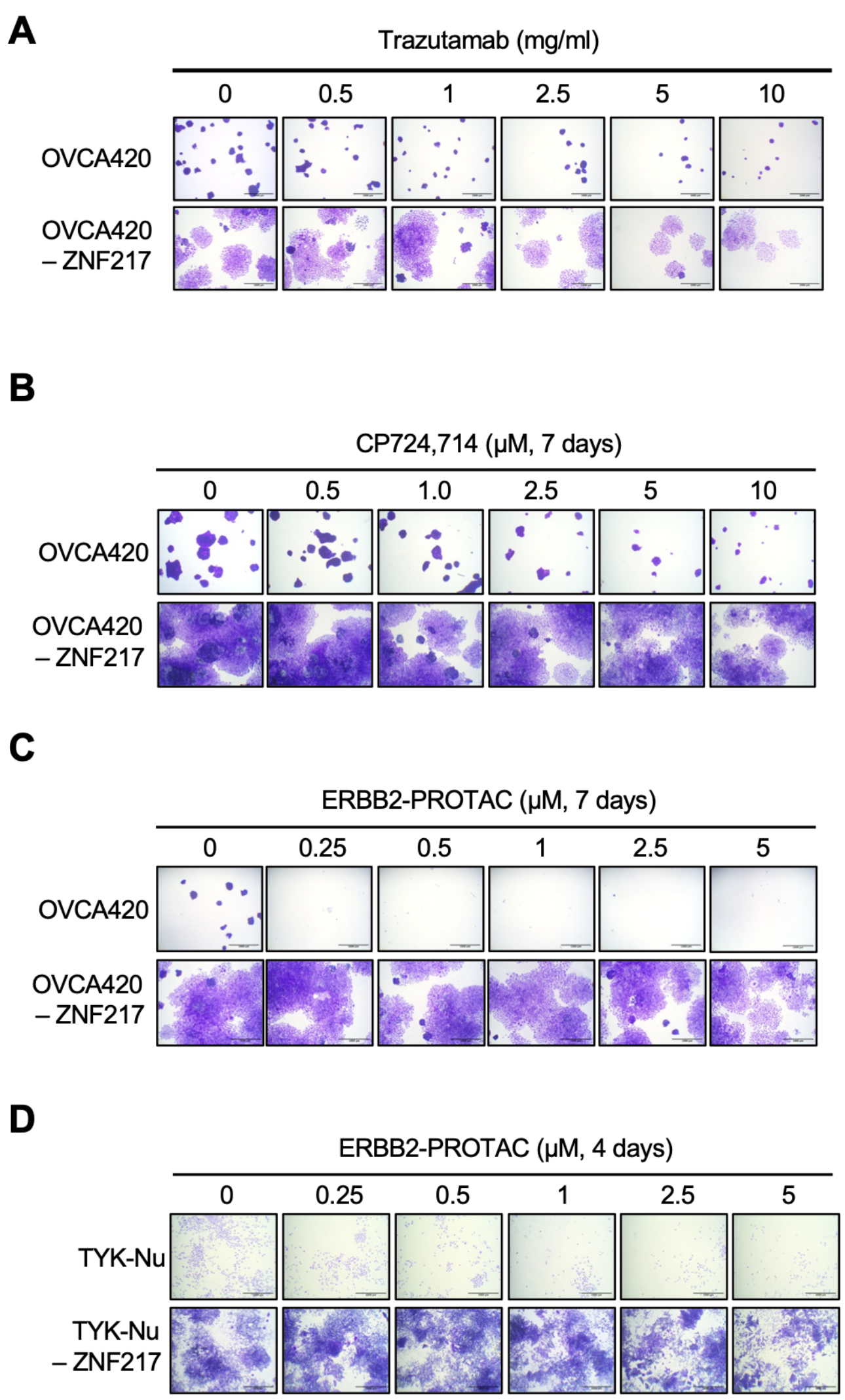

Figure S2. ZNF217-high ovarian cancer cells are more resistant to ERBB2-targeting therapeutics (A) Representative crystal violet staining showing OVCA420-ZNF217 cells are more resistant to trastuzumab (7 days treatment). **(B)** Representative crystal violet staining showing OVCA420-ZNF217 cells are more resistant to CP724,714 (7 days treatment). **(C)** Representative crystal violet staining showing OVCA420-ZNF217 cells are more resistant than control OVCA420 cells to ERBB2-PROTAC (7 days treatment). **(D)** Representative crystal violet staining showing TYK-Nu-ZNF217 cells are more resistant than control TYK-Nu cells to ERBB2-PROTAC (4 days treatment).

Fig. S3

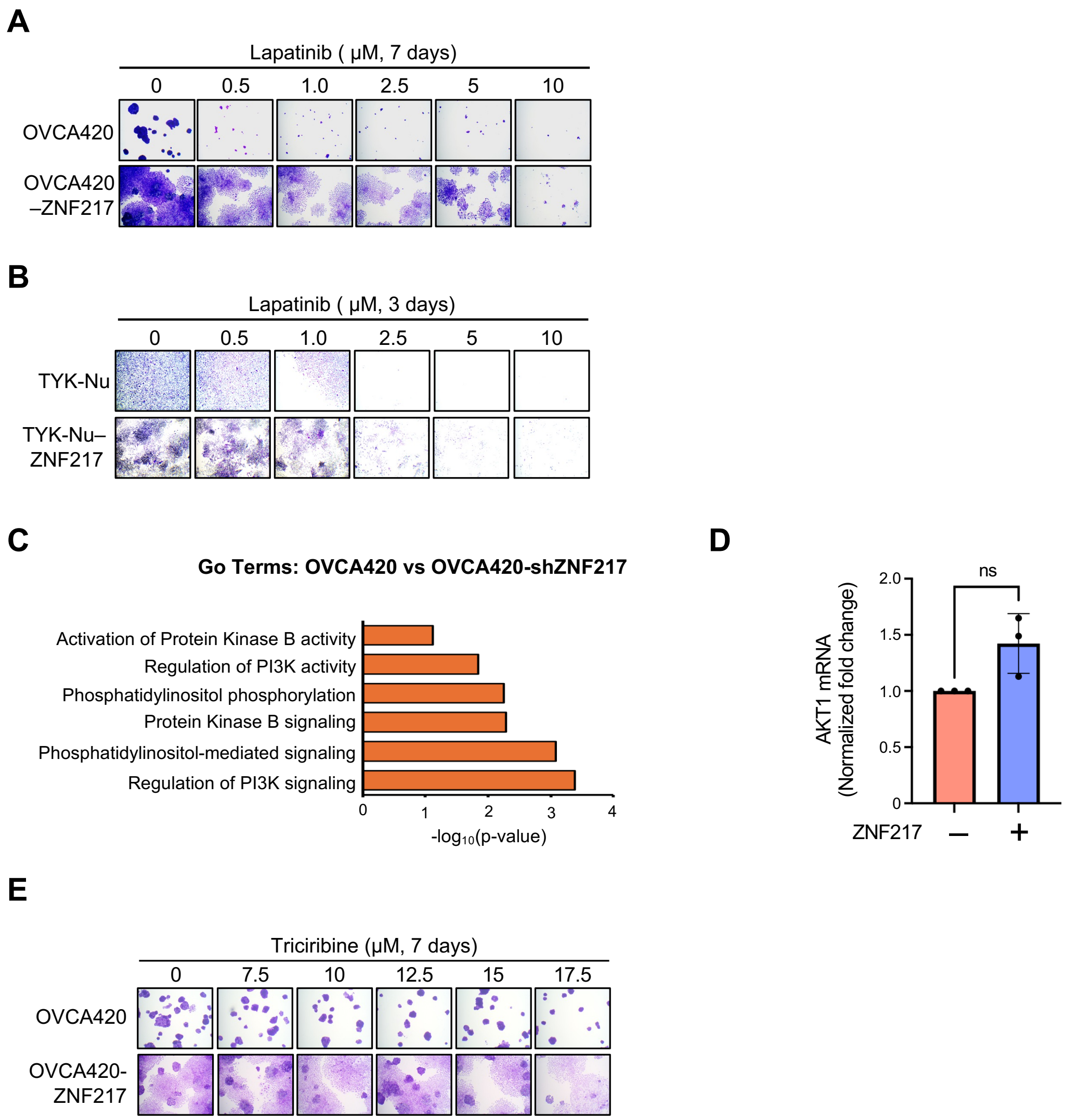

Figure S3. ZNF217-mediated resistance to anti-ERBB2 therapeutics is not dependent on EGFR and AKT signaling pathway. (A) Representative crystal violet staining showing OVCA420-ZNF217 cells are more resistant to lapatinib (7 days treatment). **(B)** Representative crystal violet staining showing TYK-Nu-ZNF217 cells are more resistant to lapatinib (4 days treatment). **(C)** Protein Kinase B signaling related Gene Ontology terms are significantly enriched in the differentially expressed gene sets in ZNF217 knockdown OVCA420 cells. **(D)** RT-qPCR analysis showing no change in AKT mRNA levels upon ZNF217 overexpression in OVCA420 cells. **(E)** Representative crystal violet staining showing that sensitivity of OVCA420 cells to long-term triciribine treatment (7 days) is not impacted by ZNF217 overexpression.

Fig. S4

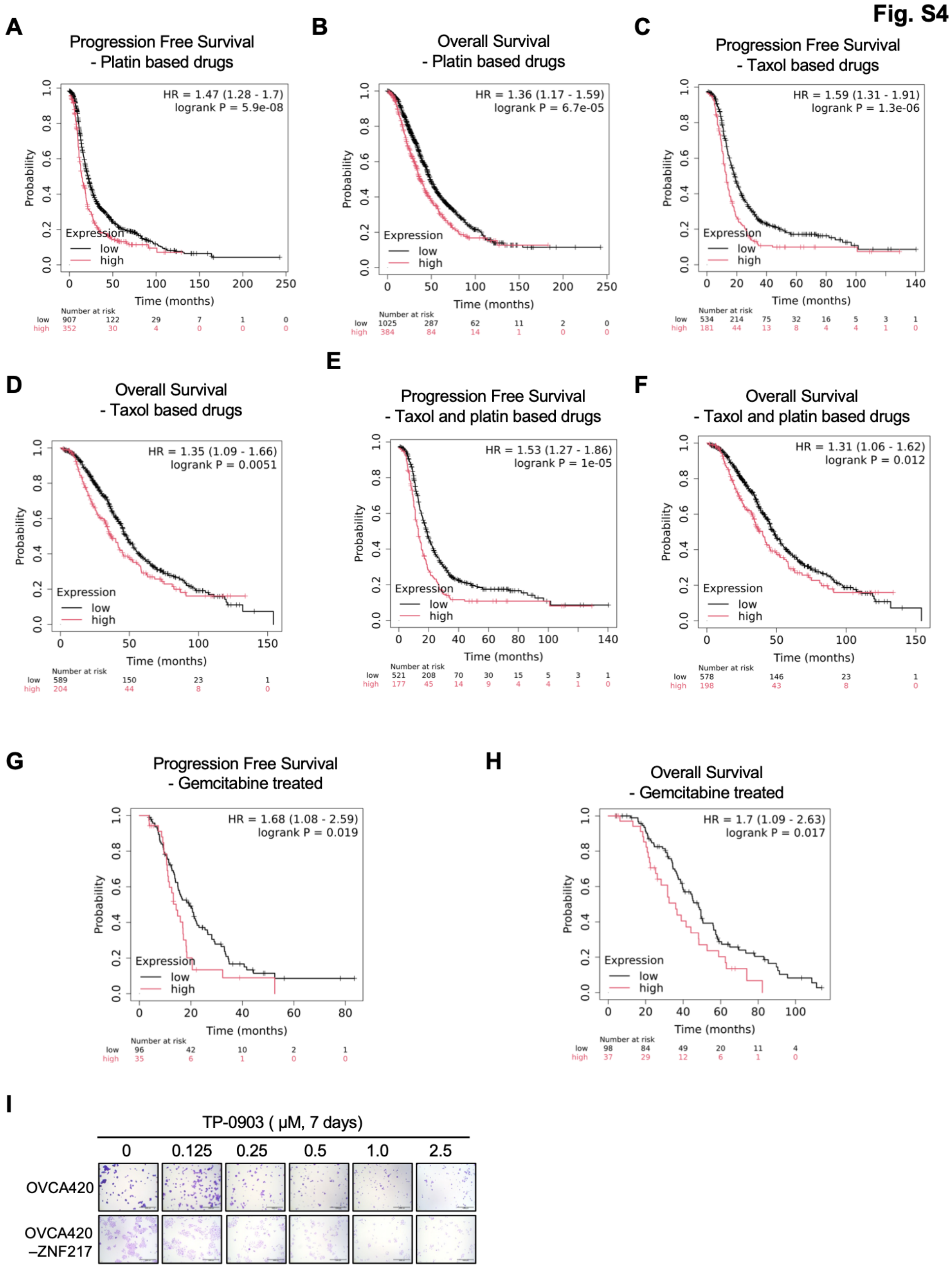

Figure S4. AXL overexpression in ovarian tumors is associated with increased resistance to multiple chemotherapeutic agents. (**A** and **B**) Clinical data from Kmplotter database show that patients with AXL-high ovarian tumors exhibit lower **(A)** progression free survival and **(B)** overall survival when treated with platin-based drugs. (**C** and **D**) Clinical data from Kmplotter database show that patients with AXL-high ovarian tumors exhibit lower **(C)** progression free survival and **(D)** overall survival when treated with taxol-based drugs. (**E** and **F**) Clinical data from

Kmplotter database show that patients with AXL-high ovarian tumors exhibit lower **(E)** progression free survival and **(F)** overall survival when treated with a combination of platin- and taxol-based drugs. **(G** and **H**) Clinical data from Kmplotter database show that patients with AXL-high ovarian tumors exhibit lower **(G)** progression free survival and **(H)** overall survival when treated with gemcitabine. **(I)** Representative crystal violet staining showing OVCA420-ZNF217 cells are more sensitive to the AXL inhibitor, TP-0903 (7 days treatment).

Fig. S5

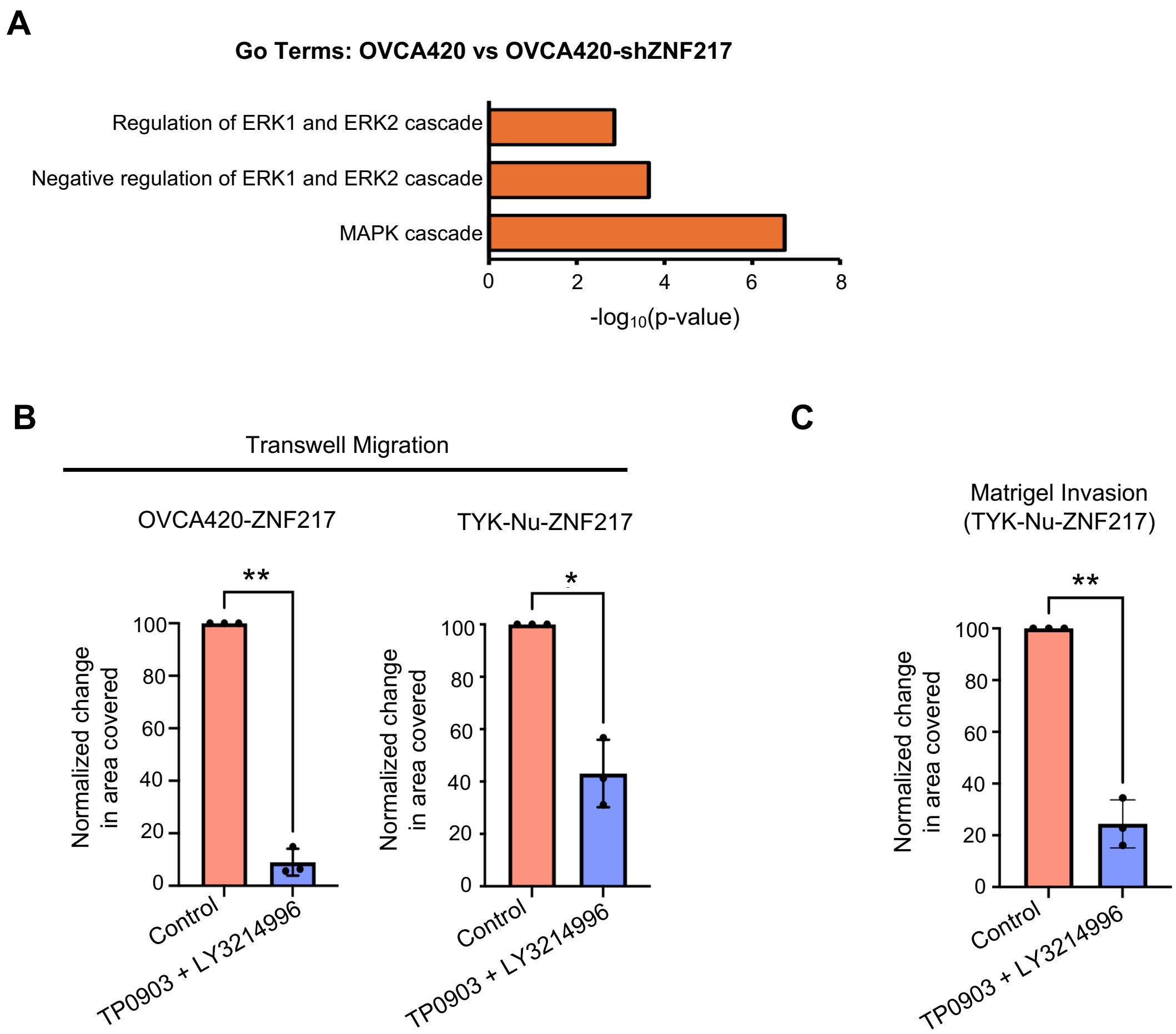

Figure S5. MAPK signaling is critical for ZNF217 driven metastatic phenotypes in ovarian cancer cells. (A) MAPK signaling related Gene Ontology terms are significantly enriched in the differentially expressed gene sets in ZNF217 depleted OVCA420 cells. **(B)** Effect of combined inhibition of AXL using TP-0903 (100 nM) and ERK using LY3214996 (1 μM) on cell migration in OVCA420-ZNF217 and TYK-Nu-ZNF217 cells using transwell migration assay. **(C)** Effect of combined inhibition of AXL using TP-0903 (100 nM) and ERK using LY3214996 (1 μM) on the ability if TYK-Nu-ZNF217 cells to invade through the matrigel.
